# Mapping iron in human heart tissue with synchrotron x-ray fluorescence microscopy and cardiovascular magnetic resonance

**DOI:** 10.1186/s12968-014-0080-2

**Published:** 2014-09-27

**Authors:** Michael J House, Adam J Fleming, Martin D de Jonge, David Paterson, Daryl L Howard, John-Paul Carpenter, Dudley J Pennell, Tim G St Pierre

**Affiliations:** School of Physics, The University of Western Australia, Crawley, Western Australia Australia; Australian Synchrotron, Clayton, Victoria Australia; NIHR Cardiovascular Biomedical Research Unit, Royal Brompton and Harefield NHS Foundation Trust, London, UK; Imperial College, London, UK

**Keywords:** Heart iron, Synchrotron X-ray fluorescence microscopy, Magnetic resonance imaging, Relaxometry, Diamond Blackfan anaemia

## Abstract

**Background:**

MRI assessment of cardiac iron is particularly important for assessing transfusion-dependent anaemia patients. However, comparing the iron distribution from histology or bulk samples to MRI is not ideal. Non-destructive, high-resolution imaging of post-mortem samples offers the ability to examine iron distributions across large samples at resolutions closer to those used in MRI. The aim of this ex vivo case study was to compare synchrotron X-ray fluorescence microscopy (XFM) elemental iron maps with magnetic resonance transverse relaxation rate maps of cardiac tissue samples from an iron-loaded patient.

**Methods:**

Two 5 mm thick slices of formalin fixed cardiac tissue from a Diamond Blackfan anaemia patient were imaged in a 1.5 T MR scanner. R_2_ and R_2*_ transverse relaxation rate maps were generated for both slices using RF pulse recalled spin echo and gradient echo acquisition sequences. The tissue samples were then imaged at the Australian Synchrotron on the X-ray Fluorescence Microscopy beamline using a focussed incident X-ray beam of 18.74 keV and the Maia 384 detector. The event data were analyzed to produce elemental iron maps (uncalibrated) at 25 to 60 microns image resolution.

**Results:**

The R_2_ and R_2*_ maps and profiles for both samples showed very similar macro-scale spatial patterns compared to the XFM iron distribution. Iron appeared to preferentially load into the lateral epicardium wall and there was a strong gradient of decreasing iron, R_2_ and R_2*_ from the epicardium to the endocardium in the lateral wall of the left ventricle and to a lesser extent in the septum. On co-registered images XFM iron was more strongly correlated to R_2*_ (r = 0.86) than R_2_ (r = 0.79). There was a strong linear relationship between R_2*_ and R_2_ (r = 0.87).

**Conclusions:**

The close qualitative and quantitative agreement between the synchrotron XFM iron maps and MR relaxometry maps indicates that iron is a significant determinant of R_2_ and R_2*_ in these ex vivo samples. The R_2_ and R_2*_ maps of human heart tissue give information on the spatial distribution of tissue iron deposits.

**Electronic supplementary material:**

The online version of this article (doi:10.1186/s12968-014-0080-2) contains supplementary material, which is available to authorized users.

## Background

Quantitative cardiovascular magnetic resonance imaging (CMR) techniques have been developed to non-invasively assess iron loading in the heart. CMR assessment of cardiac iron is particularly important for assessing transfusion-dependent anaemia patients, who can accumulate dangerous levels of iron in the heart that can eventually lead to heart failure. Chelation therapy has been successful in treating these patients, but directly monitoring heart iron loading is difficult.

Historically iron distribution has been measured and mapped using histological staining, which can provide some detailed information on iron distribution across small areas [1], but these stains are specific to the chemical speciation of iron and only semi-quantitative. Biochemical analysis of post-mortem heart samples suggests that there is a gradient in iron concentration from the epicardial to the endocardial layer [2,3]. However, these bulk-sample techniques are relatively crude, destructive, and lack detailed spatial resolution. Biopsies are invasive and potentially unreliable because of the uneven distribution of iron in the heart.

To overcome the limitations inherent in biopsied material, quantitative CMR techniques have been developed to assess iron loading in the heart. These non-invasive techniques rely on measuring the proton transverse relaxation time or T_2*_. Typically a T_2*_ image of a cross-section through the heart is generated and a region of interest in the septum selected to calculate an average T_2*_ value. A low value of T_2*_ (less than 20 ms) indicates iron overload in the heart tissue [4] and for patients with T_2*_ less than 10 ms the risk of heart failure is significantly increased [5].

While the concentration of iron has a strong effect on T_2*_, it is not the sole determinant as the distribution and size of the iron deposits and susceptibility artefacts (e.g. proximity of blood vessels) can affect the relaxation time. The potential susceptibility artefacts in vivo limit the reliable region of the heart that can be assessed using T_2*_ to the interventricular septum, a bridge of tissue separating the left and right ventricles [6,7].

Determining how closely CMR measurements represent the actual iron distribution in the human heart has been hindered by technical issues and artefacts associated with measuring the beating heart in vivo and a lack of quantitative elemental data at sufficient spatial resolution. CMR images are comprised of voxels, each voxel representing a sample volume of about 4 mm^3^ (ex-vivo), in comparison to the sample volumes of bulk biochemical methods, which are typically 150 times larger. Hence, relating CMR measurements to elemental analysis results, for example, is likely to be affected by partial volume sampling bias. In principle these errors can be minimised by making ex vivo CMR measurements on heart samples [3,8], but until recently obtaining elemental maps at a comparable resolution to CMR has been difficult.

Elemental mapping techniques based on synchrotron XFM have successfully imaged iron and other metals in human and rat brain tissue at resolutions approaching one micron [9-14]. We hypothesised that a similar elemental mapping technique applied to the heart, combined with ex vivo quantitative CMR measurements, would allow a much closer comparison and better understanding of the relationship between R_2*_, R_2_ and iron in the heart. We now present a single case history examination comparing iron maps generated by synchrotron XFM and quantitative CMR measurements.

## Methods

### Case history

The heart tissue for this case study came from a larger study of 12 patients investigating the amount and distribution of iron in the heart [8]. The 22 year old male patient had Diamond Blackfan anaemia and died of cardiac failure and pneumonia. He was transfusion dependent and received chelation therapy from the age of 10, but compliance was poor. His mean left ventricular iron concentration was 3.91 mg/g dry weight [8]. The study protocol was approved by all local research ethics committees, and local consent was obtained.

### Sample preparation

The heart was fixed in 10% neutral buffered formalin. Two slices of 5 mm thickness were cut in the ventricular short axis. The smaller slice was taken from a location closer to the heart apex and was labelled slice 7968. The larger slice was taken from a location closer to the mid-section and was labelled slice 7970.

### CMR and analysis protocols

All CMR measurements were made on Siemens 1.5 T Avanto scanner (Siemens Medical Systems, Erlangen, Germany) using a phased-array body coil. R_2_ measurements were made with the tissue samples in air. A single spin echo sequence was used to acquire R_2_ data with seven echo-times between 6 and 30 ms, a TR of 750 ms, a flip angle of 90 degrees, and two signal averages. The field of view was 90 by 120 mm and the matrix was 192 by 256 pixels. For R_2*_ measurements the heart slices were placed between Perspex plates and immersed in ultrapure water in such a way as to minimise the presence of bubbles. The temperature of the water containing the samples was 21.5°C. A multi-echo gradient echo sequence was used to acquire R_2*_ data with eight echo-times (TE) between 2.47 and 18.95 ms, a repetition time (TR) of 22 ms, a flip angle of 35 degrees, and four signal averages. The field of view was 130 by 130 mm interpolated onto a 256 by 256 matrix. Coronal slices of 4 mm thickness were acquired parallel to the top and bottom surfaces of the tissue. Using high-resolution localiser images the slices were positioned such that the majority of the slice was within the margin of the tissue.

R_2_ and R_2*_ values were calculated for each voxel using the CMR Analysis Calculator plug-in for ImageJ (v1.42). The software fits a monoexponential decay function of the form,

1 $$ \mathrm{S}\left(\mathrm{T}\mathrm{E}\right) = \mathrm{S}(0){\mathrm{e}}^{-}{{{}^{\mathrm{R}}}_{2*}}^{\mathrm{TE}}, $$

to the data, where S(0) is the initial amplitude. Voxels were excluded where the goodness of fit parameter (R^2^) was less than 0.9. Maps of R_2_ were filtered to reduce noise by using a median filter with a radius of two pixels.

### Synchrotron sample preparation and imaging

**Figure 1:**
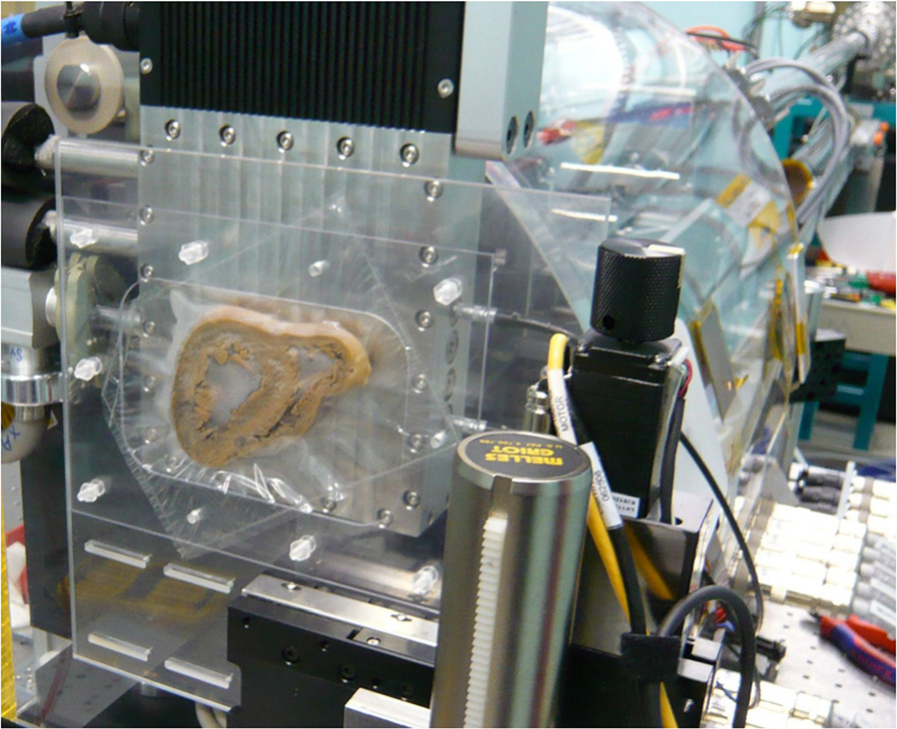
**Heart slice 7970 mounted in front of the Maia detector at the Australian Synchrotron.**

Excess formalin was removed from the samples before heat-sealing each slice inside 30 micron thick polypropylene film (Good Fellow PP301300) approximately 72 hours before the synchrotron experiment. The tissue samples were secured to a custom-made Perspex mounting plate with a thin Perspex frame (Figure 1). X-ray fluorescence data were acquired at the XFM beamline of the Australian Synchrotron [15] using the Maia 384 detector [16] and a 18.74 keV X-ray beam. The samples were mounted approximately 1 mm in front of the detector vertically in-line with the incident X-ray beam. Energy dispersive spectra were collected by scanning across the sample at a speed of approximately 6 mm s^−1^ corresponding to dwell times of 10 ms per 60 um pixel for slice 7968 and 4.2 ms per 25 um pixel for slice 7970. Scans of the entire sections took 4 to 16 hours to acquire. The event data were analyzed using the CSIRO Dynamic Analysis method [17], which enables quantitative, true-elemental images to be un-mixed from the generally complex SXRF energy spectrum. Although XFM is a quantitative technique, in this case the variable thickness and density of the specimen prevented simple quantification and so all measurements reported are relatively scaled only. In sample 7968 there was an interruption in the scanning from a technical issue which resulted in the loss of some data near the top of the sample.

### Image processing

XFM, R_2_ and R_2*_ images for sample 7970 were co-registered at the same resolution (0.5 × 0.5 mm) using ImageJ (NIH) following resampling of the high resolution XFM image to match the coarser resolution of the CMR images. To quantitatively compare the XFM and relaxometry data, 114 circular regions of interest were overlayed on the left ventricle of the co-registered XFM, R_2_ and R_2*_ maps. The R_2_ and R_2*_ values were plotted against the XFM intensity and assessed by the Pearson correlation coefficient.

### Iron analysis

The wet weight iron concentrations for the two heart slices scanned in this study were extracted from ICP-AES data acquired in a previous study on adjoining slices from the same heart [8]. The XFM iron maps were digitally overlayed onto the photographs of each sample using Photoshop CS4 (Adobe) and the approximate segments used in Carpenter et al. study [8] were digitally marked on images and annotated with the measured iron concentrations.

## Results

### XFM iron maps

**Figure 2:**
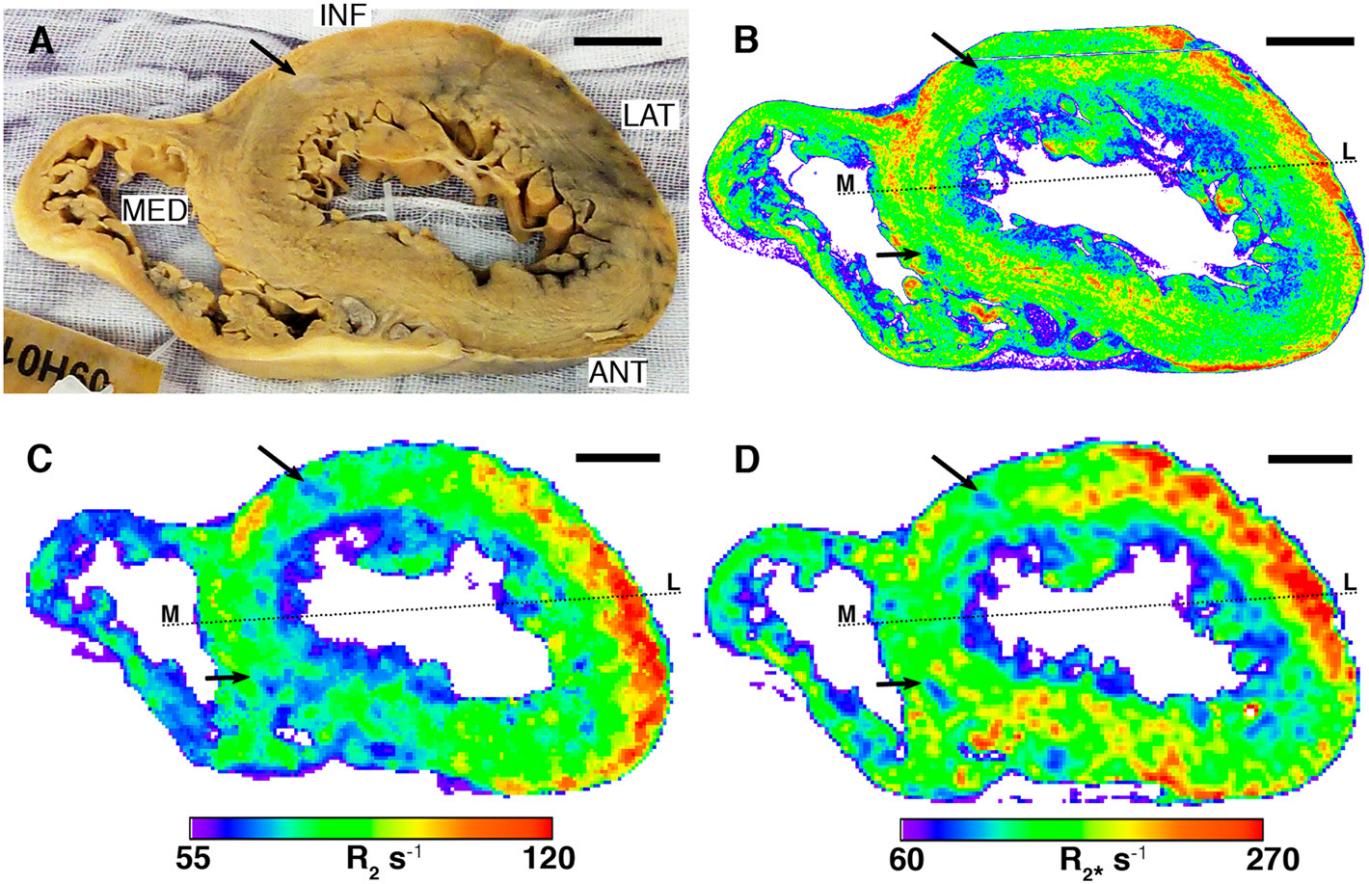
**Heart slice 7968. A)** Photo, **B)** XFM iron map, **C)** R_2_ map, **D)** R_2*_ map. Hot colours represent higher iron signal, R_2_ or R_2*_ values. Scale bar 1 cm. The dashed line indicates the position of the profile shown in Figure 4. Abbreviations: ANT anterior, INF inferior, LAT lateral, MED medial.

**Figure 3:**
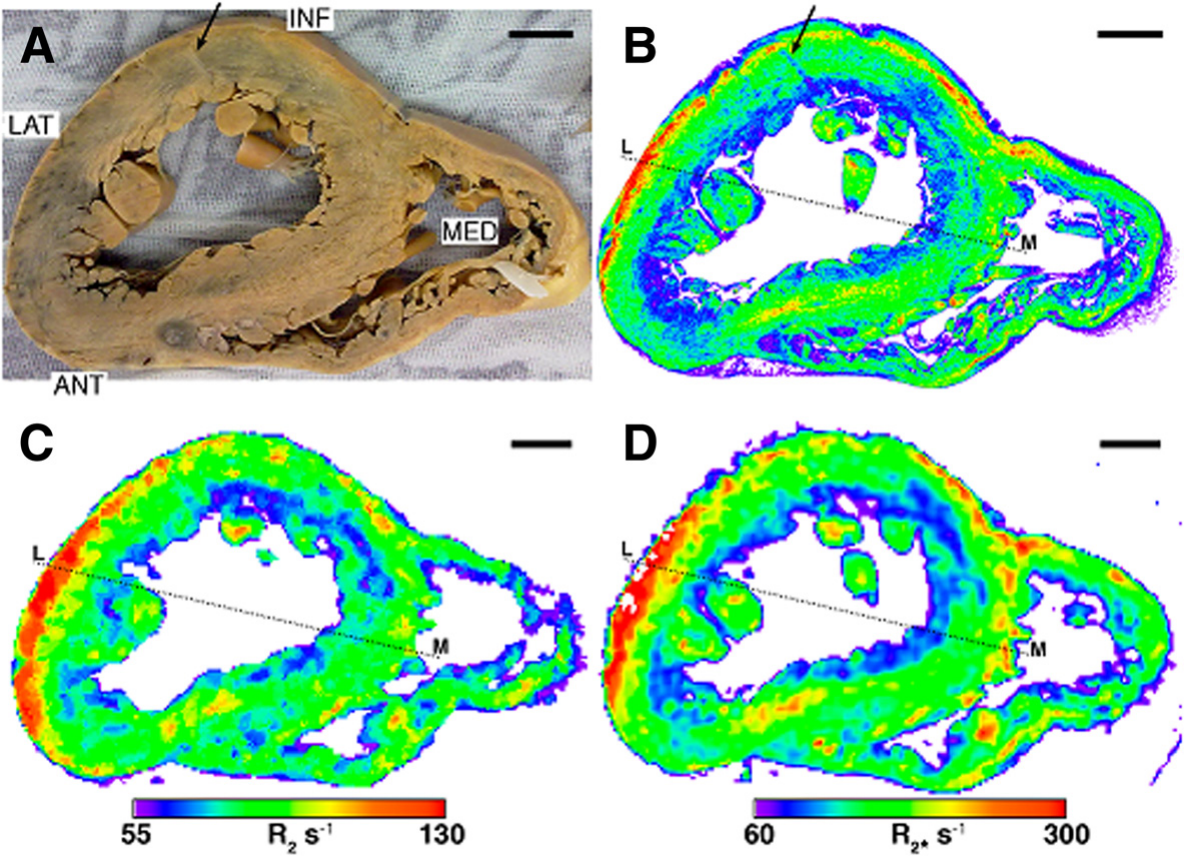
**Heart slice 7970. A)** Photo, **B)** XFM iron map, **C)** R_2_ map, **D)** R_2*_ map. Hot colours represent higher iron signal, R_2_ or R_2*_ values. Scale bar 1 cm. The dashed line indicates the position of the profile shown in Figure 5. Abbreviations: ANT anterior, INF inferior, LAT lateral, MED medial.

Photographs of the two heart samples with the corresponding XFM iron, R_2_ and R_2*_ maps are shown in Figures 2 and 3. The XFM iron maps of the thick-walled left ventricle show a strong iron signal along the lateral wall that extends to the margins of the septum for heart slice 7968 (Figure 2B), but appears to be truncated along the anterior margin of heart slice 7970 (Figure 3B). This band of high iron signal in the epicardium (outer layer) appears to partially continue into the inferior right ventricle of both samples (Figures 2B, 3B). A strong gradient in the iron distribution is apparent in the lateral wall of the left ventricle, which decreases from the outer epicardium to the inner endocardium in both heart sections.

In the interventricular septum the iron is distributed differently, particularly for slice 7968 where two discontinuous bands of higher iron are apparent (Figure 2B) within the myocardium layer. In heart slice 7970 there appears to be one dominant band of elevated iron signal, also within the myocardium (Figure 3B). The XFM iron signal is, in general, more uniform through the septum. The right ventricle of both samples shows a discontinuous band of high iron signal within the epicardium layer.

In heart slice 7968 (Figure 2B, black arrows), two oval regions of low iron signal are evident in the inferior myocardium and the anteroseptal epicardium. On the photograph of the sample (Figure 2A, black arrows), the anomaly in the inferior myocardium corresponds to a lighter coloured region of tissue about 3–4 mm across. In heart slice 7970, there is distinct linear structure that transects the inferior wall of the heart through the myocardium and endocardium layers that corresponds to a low iron signal (Figure 3B, black arrow) and a light coloured feature in the photograph of the sample (Figure 3A, black arrow). The papillary muscles also appeared to contain moderate to high iron concentrations.

### Comparison between XFM Iron Maps and CMR R_2_ and R_2*_ Maps

**Figure 4:**
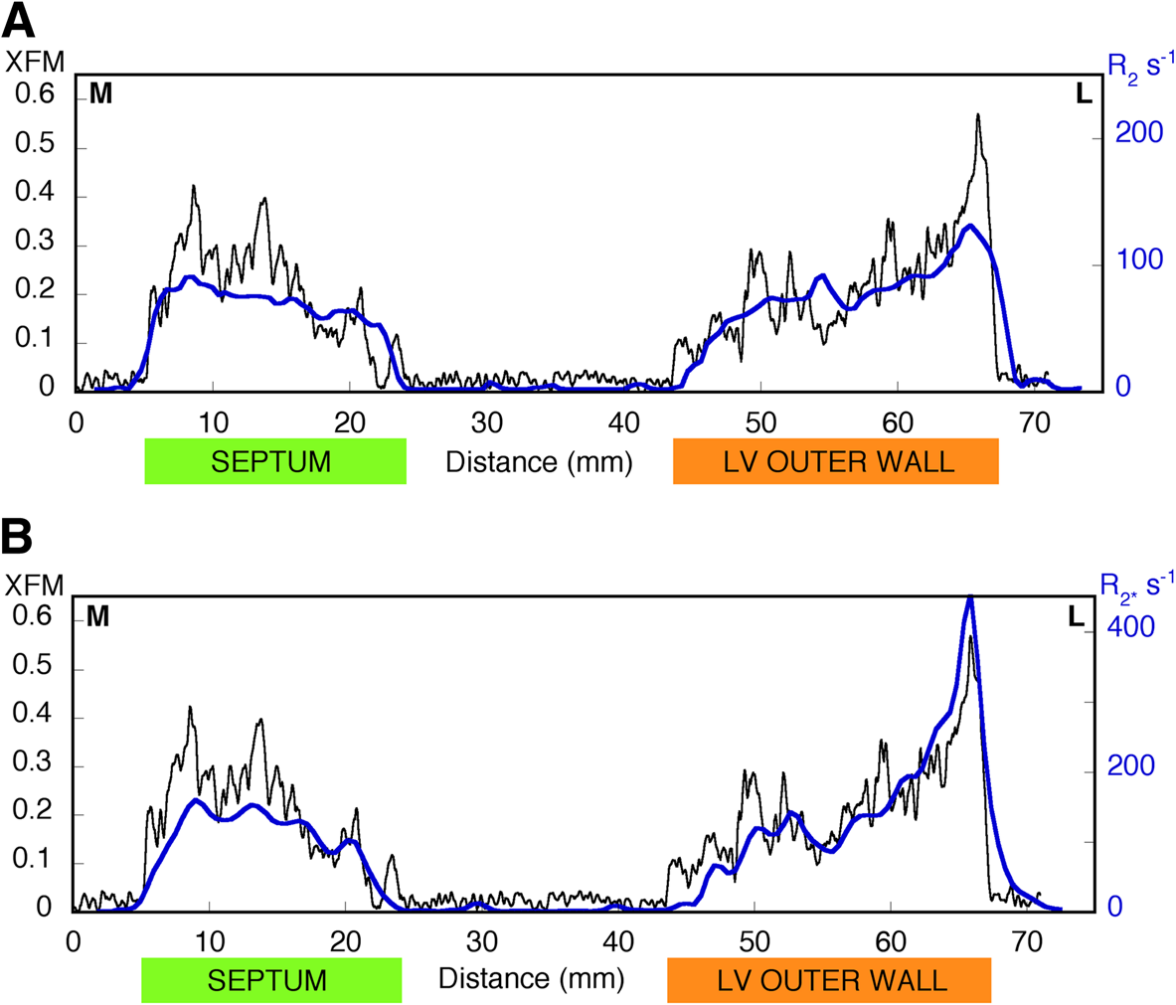
**Profile across heart slice 7968. (A)** XFM signal (black line) and R_2_ values (blue line), **(B)** XFM signal (black line) and R_2*_ values (blue line). The position of the profile is indicated by the dashed line in Figure 2. Abbreviations: M medial, L lateral, LV left ventricle.

**Figure 5:**
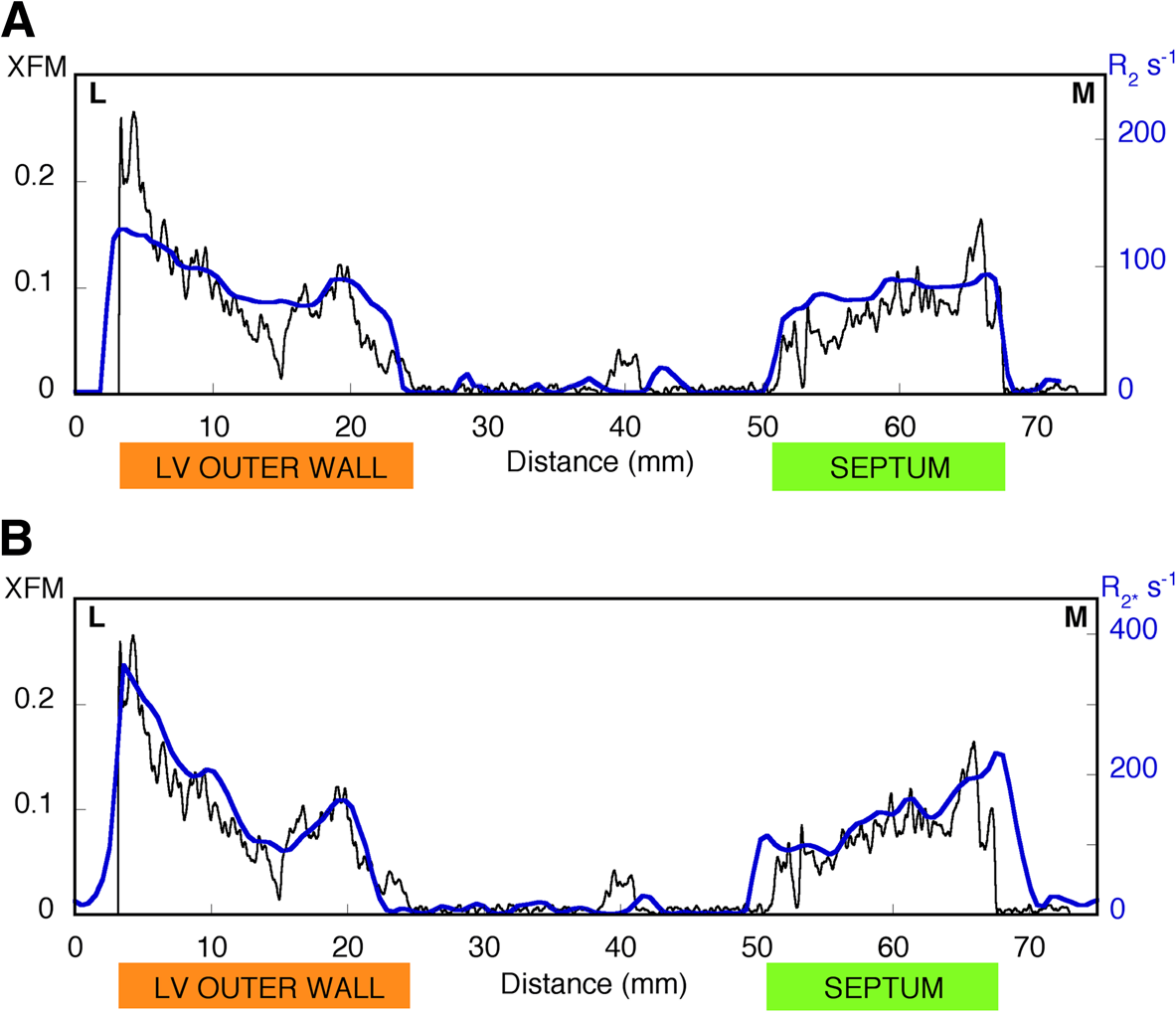
**Profile across heart slice 7970. (A)** XFM signal (black line) and R_2_ values (blue line), **(B)** XFM signal (black line) and R_2*_ values (blue line). The position of the profile is indicated by the dashed line in Figure 3. Abbreviations: M medial, L lateral, LV left ventricle.

In general, all of the macroscopic features displayed in the XFM iron maps are present in the R_2_ and R_2*_ maps (Figures 2C,D, 3C,D). In particular, the MR parameter maps for both samples have elevated R_2_ and R_2*_ values corresponding to the main high iron region in the lateral epicardium and also show the decreasing gradient from the outer to inner layer of the lateral left ventricle wall. More isolated high and low iron regions visible in the XFM maps are also apparent in the R_2_ and R_2*_ maps. Profiles across the two samples also show how the larger scale variations in XFM iron signal are largely matched by the R_2_ and R_2*_ profiles (Figures 4 and 5) and some of the finer scale features evident in the XFM profiles are also apparent in the R_2*_ profiles in particular.

**Figure 6:**
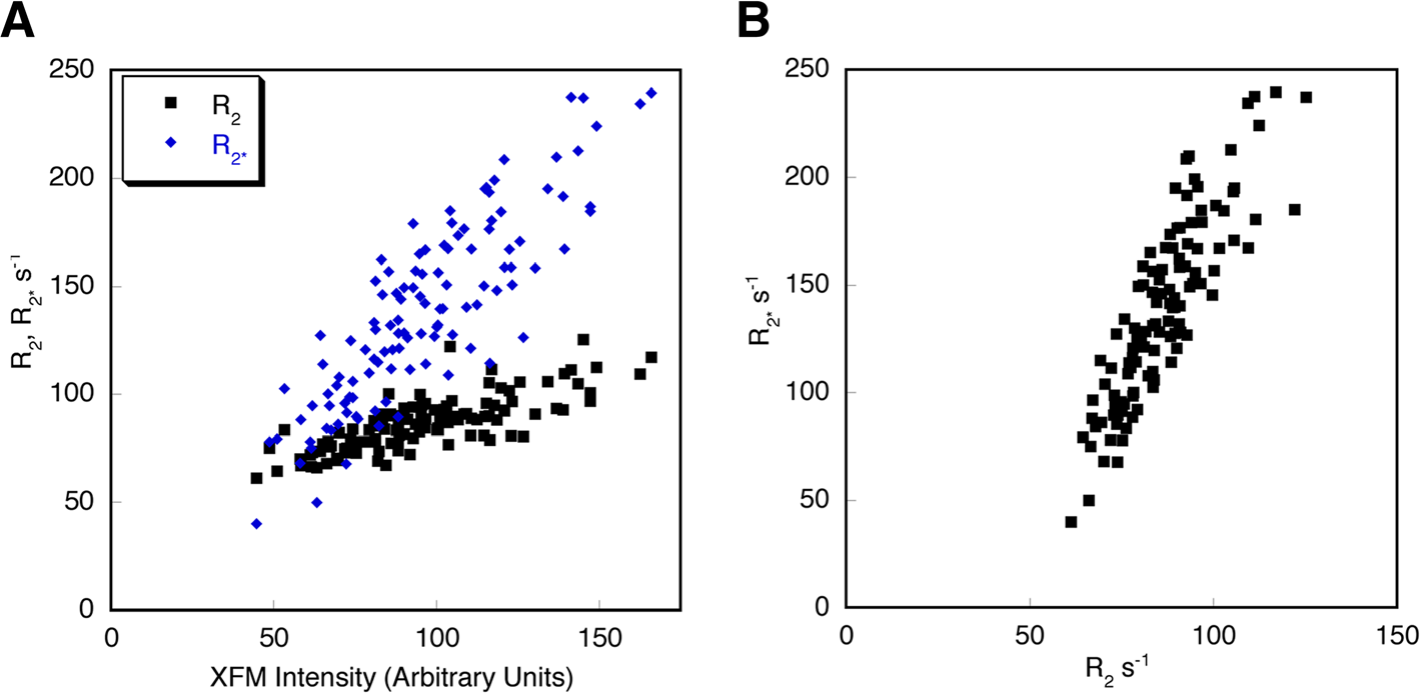
**Correlation plots for heart slice 7970.** Correlations between XFM iron intensity and R_2_, R_2*_ **(A)** and between R_2_ and R_2*_ **(B)**.

The relationships between the XFM intensity of slice 7970 and the R_2_ and R_2*_ data are shown in Figure 6. The correlation coefficients between XFM and R_2_ and R_2*_ for all samples were 0.79 (p < 0.0001) and 0.86 (p < 0.0001), respectively. The correlation coefficient between R_2_ and R_2*_ for all samples was 0.87 (p < 0.0001).

### XFM versus elemental iron concentrations

**Figure 7:**
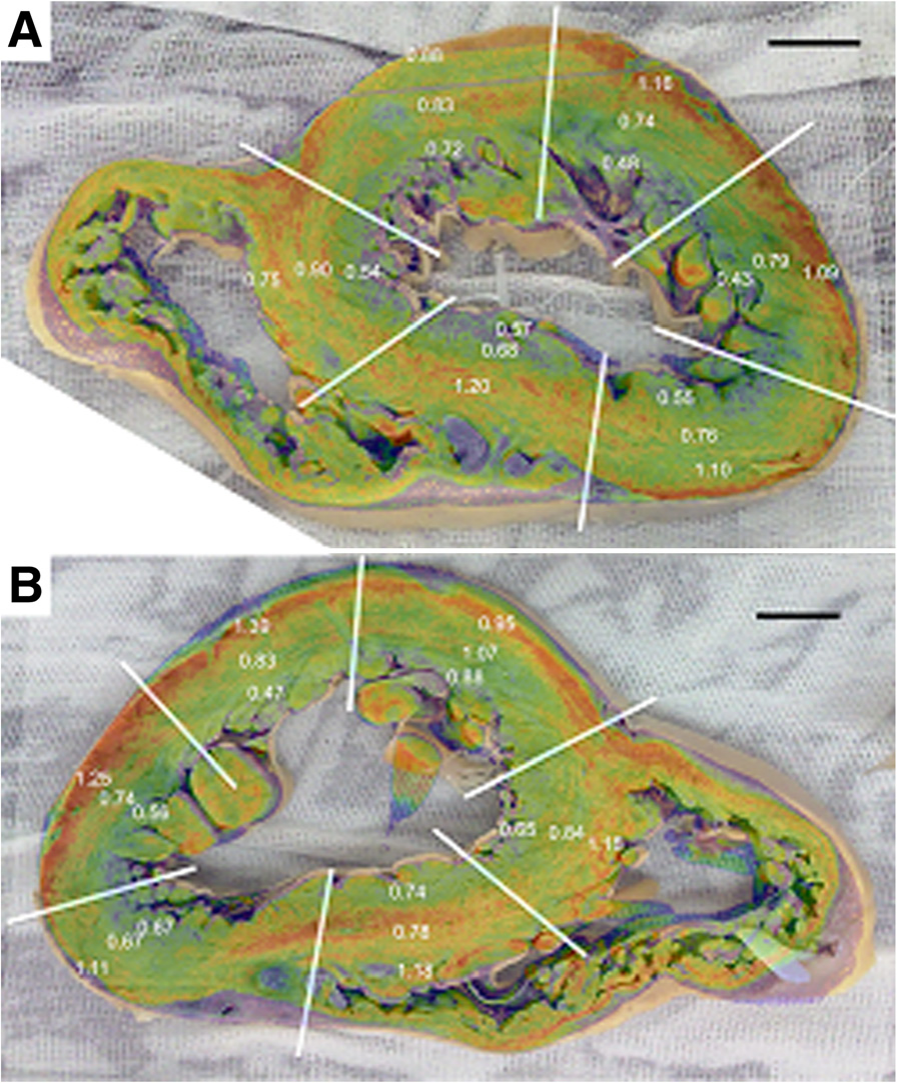
**ICP iron concentrations annotated on XFM iron maps (colour) overlayed on photographs of both heart slices. A)** sample 7968, **B)** sample 7970. The text figures in white are the iron concentrations expressed in mg of iron per gram of wet tissue. The white lines divide the slices into six segments as per the divisions used in Carpenter et al. [8]. The scale bar is 1 cm.

XFM images overlaid onto the photographs of the two samples are shown in Figure 7 annotated with the iron concentration data acquired from adjacent slices in a previous study [8]. Despite the low sampling density of the elemental analyses, features like the gradient in the left ventricle are discernable in both samples and in general the patterns in the XFM are matched by the variations in elemental iron concentrations. However, in some segments the elemental analysis appears to differ from the XFM intensity and those discrepancies are most likely to be related to the unevenness of the surface which could not be made perfectly flat (e.g. inferoseptal epicardium layer (Figure 7A 0.75 mg/g), anteroseptal myocardium layer (Figure 7B 0.78 mg/g)). The highest mean iron concentration of 1.39 mg/g corresponds to the inferior lateral epicardium in sample 6970 (Figure 7B) and in general the iron concentration of the epicardium of both samples exceeds 1 mg/g. Iron concentrations in the endocardium are typically around 0.5 mg/g for these samples.

## Discussion

To our knowledge this is the first demonstration of high-resolution synchrotron XFM elemental imaging of an entire cross section of human heart tissue and the first study to compare the distribution of XFM iron maps to quantitative CMR R_2_ and R_2*_ images. The fast scanning approach coupled with the Maia detector allowed the acquisition of XFM iron maps that displayed the internal iron distribution of the heart in great detail. Both the R_2_ and R_2*_ maps displayed a high level of correspondence to the macro-scale distribution of iron in the XFM images and profiles. On co-registered images, R_2_ and R_2*_ also showed strong linear correlations with the XFM signal.

The gradient of decreasing iron from the epicardium to endocardium we observed in XFM and transverse relaxation rates is consistent with the observations of Carpenter et al. [8] and Ghugre et al. [3]. Compilation of the iron concentrations from all the samples from this heart (Additional file 1: Figure S1) also confirm that mean iron concentrations in the epicardium are significantly higher compared to the mean iron concentrations in the myocardium and endocardium. It is not clear why iron appears to preferentially load into the lateral epicardium of this patient. Consistent with our case study, an in-vivo study on Thalassemia Major and Sickle Cell Disease patients by Ghugre et al. [18] observed elevated R_2*_ values in the lateral segment of the heart for patients with T_2*_ greater than 20 ms compared to patients with T_2*_ less than 20 ms. In contrast, the case-study of a 24 year old thalassaemic heart by Ghugre et al. [3] measured similar iron concentrations to our study, but found the outer septal layer had the highest mean iron concentration, while the lateral epicardium had the lowest iron concentration in the left ventricle epicardium layer, as measured by elemental analysis. Interestingly, the lateral epicardium in that study had the highest mean R_2*_ and R_2_, consistent with our case study. Other studies have reported segmental variation in R_2*_ for TM patients [19] consistent with the variations we observed in inferior and anterior segments. Notwithstanding the results of these workers, observations from this single case study on the distribution of iron in heart tissue may not necessarily be generalizable to other patients with cardiac iron overload.

Although a gradient in XFM iron exists in the septum of both samples, it is not as strong or consistent as in the lateral parts of the left ventricle. The images and profiles suggest that, at least for this case study, the distribution of iron, R_2_ and R_2*_ in the septal wall is more uniform overall with less extremes in the range of iron concentrations, particularly in the outer layer. The lack of a strong iron gradient in the septum potentially relates to the absence of an epicardium layer in the septum, which instead comprises a left ventricle endocardium, a mesocardium and a right ventricle endocardium [20]. However, there is still discernable structure evident in the septal region of both hearts (Additional file 2: Figure S2).

Qualitatively and quantitatively, the R_2*_ data agrees more closely with the XFM iron data than the R_2_ data. Apart from the greater sensitivity of R_2*_ to iron concentrations, as suggested by the larger regression slope between R_2*_ and XFM iron, there may be other reasons for the better agreement of the R_2*_ data. The shorter data acquisition time of the gradient echo sequence allowed 4 signals to be averaged for each gradient echo, compared to 2 averages for the spin echo data. Hence, the R_2*_ data may have benefited from higher signal to noise compared to the R_2_ data. In addition, R_2_ could potentially to be more affected by non-iron mediated relaxation mechanisms (e.g. hydration) at the iron concentrations observed in this case study.

He et al. [21] investigated in-vivo cardiac R_2_ and R_2*_ and observed that the relationship between R_2_ and R_2*_ is curvilinear. Our in-vitro data on fixed cardiac tissue, and that of Ghugre et al. [3] from fresh cardiac tissue, both show strong linear relationships between R_2_ and R_2*_ over a comparable range in R_2*_ values to He et al. [21]. While differences may arise comparing in-vivo to in-vitro measurements, another possible explanation for the discrepancy is the difference in R_2_ acquisition sequences. While the multi-echo gradient echo sequences between all three studies are similar, the R_2_ measurements in our study and Ghugre et al. [3] used a single spin echo acquisition where as He et al. [21] were restricted to a fast multi-echo spin echo acquisition to enable a short breath-hold acquisition in vivo. A multi-echo spin echo acquisition will potentially reduce the measured R_2_ relative to a single spin echo measurement as the multi-echo refocusing pulses suppress the effects of diffusion. Such a reduction in R_2_ may explain the curvilinear relationship observed by He et al. [21]. Alternatively, the simple fitting of all echo times we used in this study could, in the presence of noise, underestimate high R_2*_ values potentially masking a more curvilinear relationship. In regard to the curvilinear relationship between R_2_ and R_2*_ reported in the liver [22], we note that at relatively low iron concentrations, such as in this case study (maximum iron concentration 6 mg/g dry weight) and in Ghugre et al. [3], the relationship between R_2_ and R_2*_ is essentially linear.

It is difficult to extrapolate the CMR observations from this in-vitro study to the in-vivo measurement of cardiac T_2*_. Our study was performed on formalin fixed tissue and we reduced the magnetic susceptibility artefacts from interfaces found between living tissue and air or blood by acquiring our R_2*_ data with the sample immersed in water. In-vitro imaging also does not have to contend with the motion of the beating heart. The septal region of the heart is typically used in cardiac T_2*_ imaging for iron assessment [6,7] as it is bounded by blood on both sides and hence susceptibility variations are potentially minimised [3]. Assuming the in-vivo and in-vitro septum are likely to be the most similar in terms of their R_2*_ response, our results are consistent with R_2*_ over the whole septum being a reasonable proxy for heart iron concentration in this region.

## Conclusions

The ability to map elements in large tissue specimens using a synchrotron source can highlight new insights into fundamental biological systems and importantly provides a non-destructive technique for comparison and validation of other imaging modalities. The close qualitative and quantitative agreement between the synchrotron XFM iron maps and CMR relaxometry maps indicates that iron is a significant contributor to R_2_ and R_2*_ in these ex vivo samples and that R_2_ and R_2*_ maps of human heart tissue give information on the spatial distribution of tissue iron deposits.

### Consent

The study protocol was approved by all local research ethics committees, and local consent was obtained.

## Additional files

Additional file 1: Figure S1. ICP iron concentrations from all left ventricle epicardium, myocardium and endocardium samples for this patient’s heart. The horizontal lines indicate the mean iron concentration for each group. Additional file 2: Figure S2. Colour-shaded topography-like images of the XFM iron maps for sample 6968 (top) and 6970 (bottom). Hot colours represent higher iron fluorescence signal and the height in the z-direction shows the strength of the iron fluorescence signal. These displays highlight the general decreasing concentration of iron from the epicardium to the endocardium and also the fine-scale discontinuous concentric bands within the myocardium.
