# Supplementary figures and images for: Mapping iron in human heart tissue with synchrotron x-ray fluorescence microscopy and cardiovascular magnetic resonance
Source: J Cardiovasc Magn Reson. 2014 Sep 27;16(1):80. doi: 10.1186/s12968-014-0080-2 (PMC4177424; doi:10.1186/s12968-014-0080-2)

Fe mg/g wet weight

1.4  
1.2  
1  
0.8  
0.6  
0.4

■ Fe EPI  
◆ Fe MYO  
▼ Fe ENDO

EPICARDIUM

MYOCARDIUM

ENDOCARDIUM

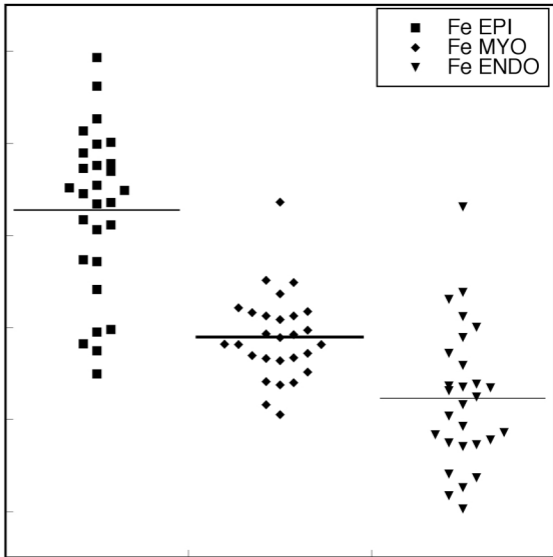

Supplement: Additional file 1: Figure S1. — ICP iron concentrations from all left ventricle epicardium, myocardium and endocardium samples for this patient’s heart. The horizontal lines indicate the mean iron concentration for each group. [file 12968_2014_80_MOESM1_ESM.pdf]

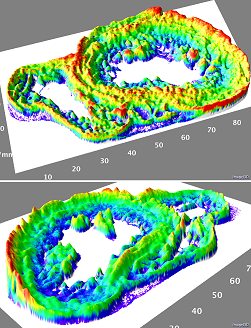

Supplement: Additional file 2: Figure S2. — Colour-shaded topography-like images of the XFM iron maps for sample 6968 (top) and 6970 (bottom). Hot colours represent higher iron fluorescence signal and the height in the z-direction shows the strength of the iron fluorescence signal. These displays highlight the general decreasing concentration of iron from the epicardium to the endocardium and also the fine-scale discontinuous concentric bands within the myocardium. [file 12968_2014_80_MOESM2_ESM.tiff]
